# Expressions of VGLUT1/2 in the inspiratory interneurons and GAD65/67 in the inspiratory Renshaw cells in the neonatal rat upper thoracic spinal cord

**DOI:** 10.1016/j.ibror.2018.08.001

**Published:** 2018-08-04

**Authors:** Makito Iizuka, Keiko Ikeda, Hiroshi Onimaru, Masahiko Izumizaki

**Affiliations:** a Department of Physiology, Showa University School of Medicine, 1-5-8 Hatanodai, Shinagawa-ku, Tokyo, 142-8555, Japan; b Department of Physiology, School of Medicine, International University of Health and Welfare, Narita Campus 4-3 Kozunomori, Narita-shi, Chiba, 286-8686, Japan; c Division of Biology, Center for Molecular Medicine, Jichi Medical University, Shimotsuke, Tochigi, 329-0498, Japan

**Keywords:** Spinal cord, Inspiratory interneuron, Renshaw cell, Neonatal rat, Vesicular glutamate transporter, Glutamic acid decarboxylase

## Abstract

Although the inspiratory spinal interneurons are thought to provide a major fraction of the excitatory synaptic potentials to the inspiratory intercostal motoneurons, this has not been confirmed. To clarify whether some inspiratory spinal interneurons are glutamatergic, we obtained whole-cell recordings from the ventromedial area of the third thoracic segments in an isolated brainstem-spinal cord preparation from neonatal rat, and the recorded cells were filled with Lucifer Yellow for later visualization. We then examined the existence of mRNA of vesicular glutamate transporters 1 and/or 2 (VGLUT1/2) by performing in situ hybridization. To discriminate the interneurons from motoneurons, we electrically stimulated the third thoracic ventral root on the recorded side, and the results verified that the antidromic spike or excitatory postsynaptic potential was not evoked. In cases in which the ventral root stimulation evoked depolarizing postsynaptic potentials, we examined the existence of glutamic acid decarboxylase 65 and/or 67 (GAD65/67) mRNA using a mixed probe to verify whether the cell was truly a Renshaw cell. The long diameter of the recorded interneurons was 22 ± 8 μm; the short diameter was 13 ± 4 μm. The interneurons' input resistance was 598 ± 274 MΩ. The Renshaw cells had similar sizes and input resistance. Six of 11 interneurons expressed VGLUT1/2, and four of five Renshaw cells expressed GAD65/67. Our findings suggest that approximately one-half of the inspiratory interneurons in the ventromedial area of the neonatal rat thoracic spinal cord are glutamatergic, and these interneurons might enhance the inspiratory intercostal motor activity.

## Highlights

• About half of the inspiratory interneurons in the ventromedial area of the third thoracic segment are glutamatergic.
• These glutamatergic interneurons may enhance the inspiratory intercostal motor activity.
• Inspiratory Renshaw cells exist in the ventromedial area of the third thoracic segments.
• Most of these Renshaw cells are GABAergic, and cause a single spike followed by ventral root stimulation at neonatal stage.

## Introduction

The basic respiratory rhythm and its motor patterns are generated by neuronal networks in the medulla that comprise the 'respiratory center' (Ezure, 1990; Bianchi et al., 1995; Onimaru et al., 1997). The respiratory center contains bulbospinal respiratory neurons as output neurons. These bulbospinal neurons regulate the motoneurons in the spinal cord that activate the various pump muscles, such as the diaphragm, external intercostal muscles, internal intercostal muscles, and abdominal muscles, in order to ventilate the lungs properly (Iizuka, 2011; Lane, 2011; Lee and Fuller, 2011). However, with the exception of the diaphragm (Lee and Fuller, 2011), it is largely unknown whether the bulbospinal respiratory neurons regulate the activity of the respiratory muscles monosynaptically or polysynaptically (Iizuka, 2011; Lane, 2011). For example, although bulbospinal inspiratory neurons are known to provide monosynaptic inputs to the intercostal inspiratory motoneurons (Davies et al., 1985a,b; Duffin and Lipski, 1987), these monosynaptic inputs were suggested to provide only a small fraction of the total depolarization needed for the discharge of the motoneurons (Davies et al., 1985b). In another study, the spike-trigger averaging of the membrane potential in an external intercostal motoneuron to the spikes of a bulbospinal inspiratory neuron was examined in 51 pairs, and monosynaptic connections between the pairs were not observed (Merrill and Lipski, 1987). Based on these results, Merrill and Lipski (1987) concluded that the bulbospinal inspiratory neurons regulate the external intercostal motoneurons via excitatory interneurons.

Extracellular and intracellular recordings have shown that many thoracic interneurons have respiratory activity (Kirkwood et al., 1988, 1993; Schmid et al., 1993; Saywell et al., 2011). The interneurons projecting to the thoracic ventral horn are distributed mainly in the contralateral medial ventral horn in the same spinal segment (Schmid et al., 1993). In their following study, they recorded from these respiratory interneurons, and described all five of the strongly modulated phasic inspiratory interneurons showed positive-going focal synaptic potential, indicating that these neurons are inhibitory (Kirkwood et al., 1993). Although the inspiratory spinal interneurons are thought to provide a major fraction of the excitatory synaptic potentials in the inspiratory intercostal motoneurons (Davies et al., 1985b; Merrill and Lipski, 1987), 'excitatory' inspiratory interneurons have not been found in the spinal cord.

The determination of the location of the excitatory inspiratory interneurons also has great importance when recovery from spinal cord injuries is considered. One of the primary causes of death among individuals who have suffered a spinal cord injury is pneumonia due to impaired respiratory function (National Spinal Cord Injury Statistical Center, 2016). It was demonstrated in a cat model that the hemisection of the spinal cord caused an immediate reduction in ipsilateral intercostal nerve activity below the injury site, but intercostal inspiratory activity recovered within a few days (Kirkwood et al., 1984), and in rats it returned within a few weeks (Dougherty et al., 2012; Zimmer et al., 2015). Based on these findings, it has been proposed that the intercostal muscles make a significant contribution to respiratory recovery after chronic cervical spinal cord injury (Dougherty et al., 2012), and plastic changes of the excitatory inspiratory interneurons in the spinal cord are suspected (Zimmer et al., 2015). Thus, the existence of the excitatory inspiratory interneurons in the spinal cord should be confirmed first.

It is well documented that the rostral part of the rib cage muscles of mammals shows larger inspiratory activity (De Troyer et al., 2005). Similarly, in an isolated brainstem spinal cord preparation from neonatal rat, the ratio of the thoracic inspiratory motor activity to the expiratory activity was larger in the rostral thoracic segment, suggesting that the neuronal mechanisms that generate the rostrocaudal gradient remain intact (Iizuka, 2004). A more recent study of a neonatal rat model showed that the inspiratory depolarizing optical signals in the motoneuron and interneuron area was larger in the more rostral thoracic spinal cord when the preparation was stained with a voltage-sensitive dye in the isolated brainstem-spinal cord preparation (Iizuka et al., 2016).

We hypothesized that some of the inspiratory interneurons in the rostral thoracic cord are excitatory and enhance the inspiratory motor outputs. Here we examined the existence of mRNA of vesicular glutamate transporters 1 and/or 2 (VGLUT1/2) by performing in situ hybridization with these inspiratory interneurons.

During the present experiment, some of the recorded cells showed excitatory postsynaptic potentials after electrical stimulation to the ventral root. Renshaw cells receive excitatory inputs from the motoneuron axon collaterals and exert a recurrent inhibition of synergist motoneurons (Alvarez and Fyffe, 2007). Although Renshaw cells are well-characterized inhibitory interneurons and have been studied especially in the lumbar spinal cord in relation to the regulation of locomotor activity (Nishimaru et al., 2006), the Renshaw cells and their location in the thoracic segments have not been fully examined (Kirkwood et al., 1981; Saywell et al., 2013). We therefore investigated the existence of mRNA of glutamic acid decarboxylase 65 and/or 67 (GAD65/67) on the recorded cells, which received depolarizing postsynaptic potentials evoked by ventral root stimulation, to verify whether these cells are truly Renshaw cells.

## Methods

### Ethical approval

This study was approved by the Animal Research Committee of Showa University, which operates in accordance with the Japanese Government's Law No. 105 for the care and use of laboratory animals.

### Brainstem spinal cord preparation

Wistar rats (n = 23), 0–2 days of age, were deeply anesthetized with isoflurane until their nociceptive reflexes were abolished. The cerebrum was then quickly removed by transection at the intercollicular level, and the brainstem and spinal cord were isolated as described (Suzue, 1984; Onimaru et al., 1988). The brainstem was rostrally decerebrated between the 6th cranial nerve roots and the lower border of the trapezoid body. The spinal cord was cut at the level between the third and fourth thoracic (T3, T4) ventral roots.

**Fig. 1:**
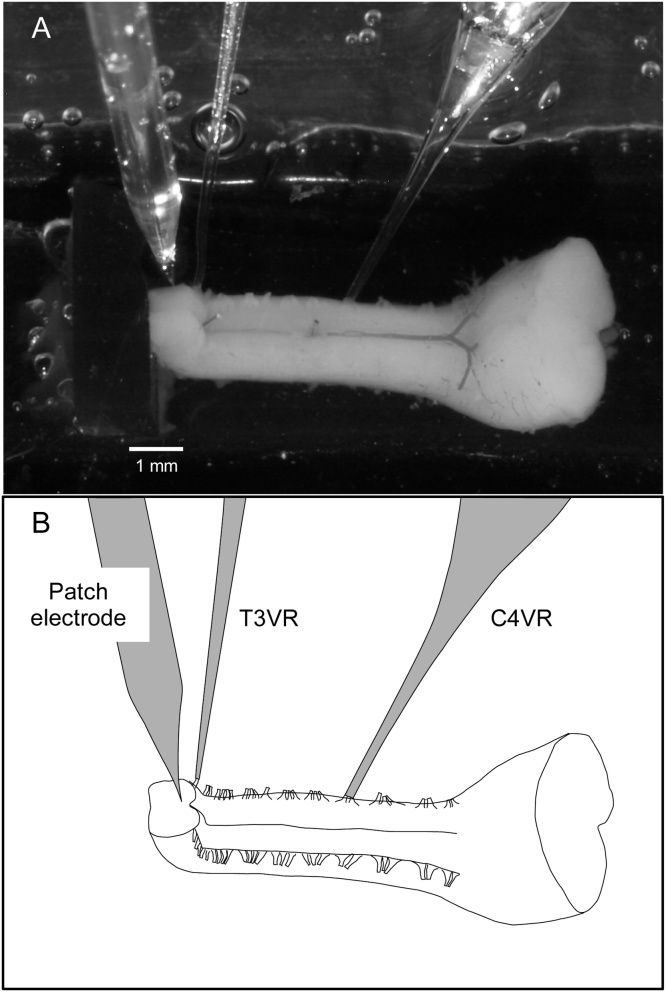
Experimental arrangement. A: Photograph of a preparation. The preparation was placed with the ventral surface up and bent ventrally at around T1 to turn the section upward, and was pinned at the midline of C5 and T1 on an L-shaped silicone rubber plate (approximately 1-mm thick, 4–6 mm × 20–25 mm with a 2.5-mm-high vertical wall). The fourth cervical ventral root (C4VR) and the third thoracic ventral root (T3VR) were incorporated into glass suction electrodes. A glass electrode for the whole-cell patch-clamp recording was inserted from the surface of the cross-section. B: Schematic drawing of panel A.

As shown in Fig. 1, the brainstem-spinal cord preparation was placed with the ventral surface up and bent ventrally at around T1 to turn the section upward, and was pinned at the midline of C5 and T1 on an L-shaped silicone rubber plate. The preparation was then moved to a 2.5-ml perfusion chamber and superfused continuously at 2–3 ml/min with modified Krebs solution consisting of (in mM): 124 NaCl, 5.0 KCl, 1.2 KH_2_PO_4_, 2.4 CaCl_2_, 1.3 MgCl_2_, 26 NaHCO_3_, 30 glucose, and equilibrated with 95% O_2_ and 5% CO_2_, pH 7.4, at 25°–27 °C.

### Whole-cell patch-clamp recordings

The membrane potentials and input resistances of neurons in the ventromedial region of the section at the third thoracic spinal cord were recorded by a blind whole-cell patch-clamp method (Onimaru and Homma, 1992). The electrodes (inner tip diameter, 1.2–2.0 μm; resistance, 4–8 MΩ) were filled with the following pipette solution (in mM): 130 K-gluconate, 10 EGTA, 10 HEPES, 2 Na_2_-ATP, 1 CaCl_2_ and 1 MgCl_2_, at pH 7.2–7.3 adjusted with KOH.

The membrane potentials were recorded with a single-electrode voltage-clamp amplifier (CEZ-3100; Nihon Kohden, Tokyo) after compensation for series resistance (20–50 MΩ) and capacitance. Neurons were sought by advancing the electrode into the spinal cord while monitoring amplified extracellular signals with a sound monitor. Before the whole-cell patch-clamp recording, we checked the cell firing during the inspiratory phase extracellularly. While the firing rate was often increased by advancing the electrode, subtle retraction enable us to obtain the stable firing pattern. When a target neuron was found, slightly negative pressure was applied. At the same time, the formation of a GΩ seal was monitored by applying a hyperpolarizing current pulse (0.1 nA, 30 msec, 1 Hz).

After maintaining a GΩ seal (>1 GΩ) for several minutes at a pressure around 0 cmH_2_O, we applied brief large negative pressure (about 40 cmH_2_O) to rupture the patch membrane. When the whole-cell recording was obtained, the pressure was returned to approximately 0 cmH_2_O. Since in some cells the firing rate became vigorously increased when the period of the whole-cell recording was achieved, we injected a negative current until the firing rate was decreased to a level similar to the levels observed during the extracellular recording obtained before the whole-cell recording. The resting membrane potential was measured at this condition. When a > −0.1 nA current injection was necessary to maintain the firing rate, the electrode was pulled out and the data were discarded. Input resistance was measured using Ohm's law with a constant negative current injection (from −50 to −100 pA, 0.5 s).

For the histologic analysis of the recorded cells, the electrode tips were filled with 0.5% Lucifer Yellow fluorescent stain (lithium salt; Sigma-Aldrich, St. Louis, MO). To discriminate the recorded neuron as a motoneuron, Renshaw cell, or other interneuron, we attached a glass capillary suction electrode to the T3 ventral root on the side where the patch-clamp electrode was inserted, and we electrically stimulated the T3 ventral root (0.2 msec duration, up to 10 V). When the spike potential was evoked with short latency without any postsynaptic potentials, we considered the recorded neuron a motoneuron. However, since the electrode was inserted only in the ventromedial region, the antidromic spike potential was rarely evoked by the ventral root stimulation. This was in good agreement with our previous study's finding that depolarizing optical signals were evoked by ventral root stimulation, and thus the antidromic activation of motoneurons was restricted in the middle area between the lateral edge and the midline of the spinal cord (Iizuka et al., 2016). When short-latency depolarizing postsynaptic potentials were observed, we considered the recorded neuron a Renshaw cell. Neurons in which the electrical stimulation to the T3 ventral root did not cause antidromic spike potentials or the excitatory postsynaptic potential were considered interneurons.

Inspiratory motoneuron activity was monitored at the 4th cervical (C4) ventral root with a glass capillary suction electrode. This C4 activity is known to synchronize with discharges of phrenic nerves, which are derived from the C4 and C5 ventral roots (Suzue, 1984). We also monitored the T3 ventral root activity, except during the electrical stimulation mentioned above. The ventral root activities were amplified (×2000) and band-pass filtered (0.5–1000 Hz). Each obtained electrical signal was digitized at 4 KHz (Powerlab, ADInstruments, Sydney, Australia) and stored on a personal computer using Chart v7.0/s software.

We analyzed the firing characteristics of the recorded cells related to the C4 inspiratory bursts, using 20 consecutive respiratory cycles by Spike2 software (CED, Cambridge, England). We defined the inspiratory phase as follows. The C4 activity was digitally high pass-filtered (0.01 s), fully rectified and smoothed (time constant, 0.01 s). The threshold level was set at the mean +4 fold of the standard deviation of the obtained wave during the expiratory phase (5 s). The rising and falling thresholds were defined as the onset and offset of the inspiratory phase, respectively. The measured parameters were the half-width of the spike potential, the peak firing frequency during the inspiratory phase, and the number of firings per single inspiratory burst. The half-width was measured using an averaged spike of the first spike during each inspiratory burst.

### In situ hybridization

We performed in situ hybridization followed by immunofluorescence experiments on spinal cord sections. After the electrophysiological analyses, the spinal cord was isolated and further fixed at 4 °C in fixation solution for 1–2 h. Samples were immersed in 18% sucrose/phosphate-buffered saline (PBS), embedded in optimal cutting temperature (OCT) compound (Sakura Finetek, Torrance, CA), frozen on dry ice, and cut into 30- or 12-μm-thick cryosections. The in situ hybridization was performed essentially as described (Ikeda et al., 2013), with isoform-specific digoxigenin-UTP (Roche Diagnostics, Basel, Switzerland)-labeled riboprobes for VGLUT1, VGLUT2, GAD65/GAD67, and glycine transporter 2 (GLYT2) at 50 °C.

Partial cDNAs of mouse VGLUT1 (nucleotide nos.1437–2159 of BC054462) and mouse VGLUT2 (nucleotide nos. 1805–2386 of BC038375) were obtained by reverse transcription-polymerase chain reaction (RT-PCR) using mouse brain total RNA, subcloned into pGEM-T easy Vector (Promega, Madison, WI), and confirmed by sequencing. Plasmid templates for riboprobes of GAD65/67 and GLYT2 were kindly provided Drs. Stanley Watson and Ilan Kerman and Dr. Ikuko Tanaka, respectively, as described (Onimaru et al., 2014). Proteinase K (1 μg/ml) was applied for 2 min at 26 °C.

Signals were detected using an anti-digoxigenin antibody conjugated with alkaline phosphatase (Roche) and NBT/BCIP (Roche) for chromogen. Signal detection was followed by immunofluorescence staining using rabbit anti-Lucifer yellow (1:400 dilution, Molecular Probes/Invitrogen) and Alexa Fluor 488 anti-rabbit IgG (1:1000 dilution, Molecular Probes/Invitrogen) as described (Ikeda et al., 2013). For nuclear staining, we used 4,6-diamidino-2-phenylindole (DAPI, Sigma). Images of immunofluorescent samples were obtained with 20× or 10× objectives on a conventional fluorescence microscope (BX60, Olympus Optical) with a digital camera (DS-Fi1, Nikon) at a resolution of 1280 × 960 pixels.

### Data analysis

To examine the sizes of the soma, we measured the longer and shorter diameters. When we made a rhombus using the longer and shorter diameters as diagonals, the edge of most of the cells seemed to protrude from the rhombus. When a rectangle was made using the longer and shorter diameters as longer and shorter sides, the rectangle was clearly larger than the cell. Although the cells had various shapes, we found that the combination of the longer and short diameters can be informative. We therefore calculated the area of each ellipse by using the longer and shorter diameters. Values are presented as the mean ± standard deviation. Statistical difference between two groups were examined using the Mann-Whitney *U* test The level of statistical significance was set at p < 0.05.

## Results

Whole-cell recordings were obtained from 23 inspiratory-related neurons. In 16 of the 23 neurons, neither action potentials nor synaptic potentials were evoked by the electrical stimulation to the third thoracic ventral root (T3VR). In seven of the 23 neurons, the electrical stimulation to the T3VR caused the depolarizing synaptic potential and the following spikes. We treated these seven neurons as Renshaw cells, and the other 16 neurons were treated as inspiratory interneurons.

### The inspiratory interneurons

In 11 of the 16 inspiratory interneurons, the existence of mRNA of vesicular glutamate transporters 1 and 2 (VGLUT1 and VGLUT2) was examined by in situ hybridization. Although the in situ hybridization was performed on 12-μm-thick cryosections and we examined the existence of VGLUT1 and VGLUT2 mRNA separately in the first to third preparations, the major part of the soma was in either section, making it difficult to examine the existence of VGLUT1 and VGLUT2 mRNA separately. Of these three preparations, two were VGLUT1-positive and the third was VGLUT2-positive at least. In the other eight preparations, we examined the existence of VGLUT1 and VGLUT2 mRNA at the same time using a mixed probe. In total, six of the 11 neurons were VGLUT-positive inspiratory interneurons.

**Fig. 2:**
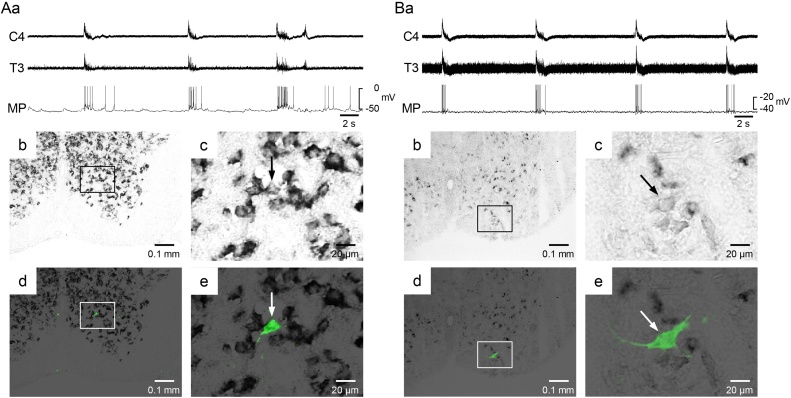
VGLUT-positive inspiratory interneurons in the third thoracic cord. A,B: A VGLUT-positive inspiratory interneuron. Aa, Ba, recordings obtained from the fourth cervical and third thoracic ventral root (C4, T3) and the membrane potential of the inspiratory interneuron (MP). Ab and Bb are fluorescence images. The recorded neuron was stained by Lucifer yellow (green). Ac and Bc are higher magnifications of the highlighted square in panels Ab and Bb. The VGLUT-positive cells were stained with black. Ad, Ae, Bd, Be: Bright-field images of the same areas as Ab, Ac, Bb, Bc, respectively. White and black arrows indicate the recorded neuron.

In the example shown in Fig. 2A, the soma size was relatively small (longer and shorter diameters: 15.30 and 9.66 μm), and this interneuron also showed many excitatory synaptic potentials during the expiratory phase, and fired occasionally. In another example (Fig. 2B), the soma size was relatively large (longer and shorter diameters: 34.62 and 15.30 μm), and this neuron showed small synaptic noise during the expiratory phase and brisk firing during the inspiratory phase.

**Fig. 3:**
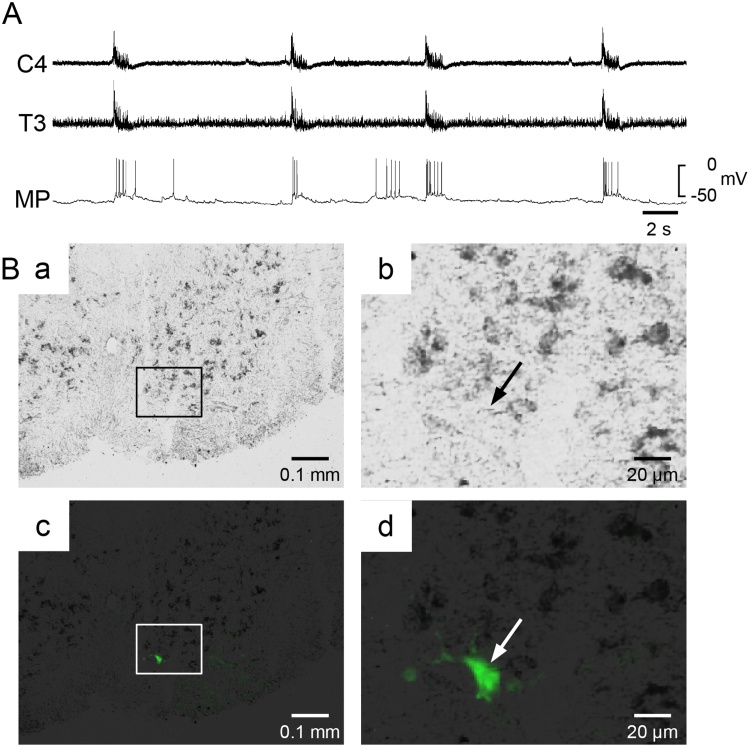
VGLUT-negative inspiratory interneuron in the third thoracic cord. A: Recordings obtained from C4 and T3 thoracic ventral root, and membrane potential of the inspiratory interneuron. Ba, b: Fluorescence images. Bc, d: Bright-field images of the same areas as Ba and b. Bb, d: Higher magnifications of the highlighted square in panels Ba, c. White and black arrows indicate the recorded neuron. Since the VGLUT-positive cells were stained with black, the recorded neuron was negative.

A representative example of a VGLUT-negative inspiratory interneuron is shown in Fig. 3. The longer and shorter diameters were 23.35 and 12.08 μm, respectively. As summarized in Table 1, there was no significant difference in the long diameter, short diameter, or ellipse area between the VGLUT-positive and -negative inspiratory interneurons. From these anatomical parameters, it was difficult to discriminate the VGLUT-positive interneurons from the negative interneurons.

**Table 1:** Anatomical and electrophysiological characteristics of the inspiratory interneurons and Renshaw cells.

Type	n	Long dia. (μm)	Short dia. (μm)	Ellipse area (μm^2^)	Resting membrane potential (mV)	Input resistance (MΩ)	Half-width of spike (ms)	No. of Firing (fire/insp. burst)	Max. Firing Freq. (Hz)
Inspiratory interneurons									
VGLUT-positive	6	19.9 ± 7.8	12.3 ± 3.4	201.8 ± 122.0	−49.8 ± 7.9	628.0 ± 291.9	2.38 ± 0.60	5.32 ± 4.74	13.84 ± 10.85
VGLUT- negative	5	19.3 ± 6.5	11.4 ± 2.3	178.5 ± 78.0	−57.5 ± 7.5	839.2 ± 208.4	2.41 ± 0.41	4.93 ± 3.46	21.66 ± 19.37
Not tested	5	28.3 ± 6.4	15.8 ± 3.8	362.0 ± 152.3	−48.4 ± 4.8	405.8 ± 145.2	2.68 ± 0.8	4.55 ± 3.72	20.32 ± 23.56
Total	16	22.3 ± 7.7	13.1 ± 3.5	244.6 ± 139.8	−51.8 ± 7.6	598.1 ± 274.4	2.48 ± 0.59	4.96 ± 3.80	18.31 ± 17.33

Renshaw cells									
GAD-positive	4	26.4 ± 9.4	12.3 ± 1.2	259.9 ± 118.3	−50.9 ± 9.2	673.5 ± 177.2	2.57 ± 0.35	6.16 ± 4.65	36.81 ± 29.14
GAD-negative	1	30.6	12.1	290.2	−41.2	326.1	2.26	1.4	3.9
GLYT2-positive	1	27.4	12.9	277.0	−50.1	317.8	1.87	3.0	7.3
Not tested	1	25.8	10.5	211.8	−43.0	304.7	2.49	9.0	28.3
Total	7	27.0 ± 6.9	12.1 ± 1.1	259.8 ± 87.1	−48.3 ± 7.8	571.5 ± 256.3	2.41 ± 0.36	5.43 ± 4.11	26.67 ± 25.35

The measured firing characteristics during the inspiratory phase are summarized in Table 1. In the three neurons shown in Fig. 2A,B and Fig. 3, the peak firing frequency during the inspiratory phase was 10.0 ± 4.8, 26.5 ± 4.5, and 22.8 ± 12.5 Hz, and the mean number of firings per inspiratory phase was 4.2 ± 2.1, 5.7 ± 1.2, and 5.6 ± 1.7, respectively. The peak firing frequency was dependent on the cell, ranging from 0.8 to 26.5 Hz in the VGLUT-positive neurons and from 4.5 to 54.1 Hz in the VGLUT-negative neurons. The number of firings per inspiratory burst ranged from 1.1 to 14.3 in the VGLUT-positive neurons and from 2.0 to 10.6 in the VGLUT-negative neurons. The half-width of the spike ranged from 1.72 to 3.13 msec in the VGLUT-positive neurons and from 2.06 to 3.09 in the VGLUT-negative neurons. There were no significant differences in any of the firing characteristics or the resting membrane potential between the VGLUT-positive and -negative neurons.

### The Renshaw cells

**Fig. 4:**
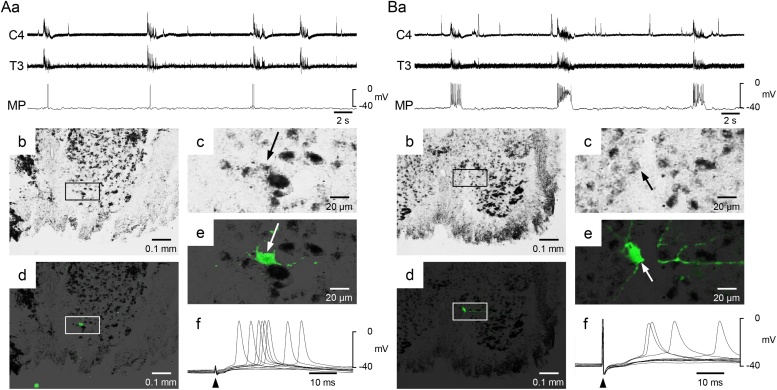
Renshaw cells in the third thoracic cord. A,B: Two examples of Renshaw cells. Aa, Ba: Recordings obtained from the fourth cervical and third thoracic ventral root (C4, T3) and the membrane potential of the Renshaw-like cell (MP). Ab, Bb: Fluorescence images. The recorded neuron was stained by Lucifer yellow (green). Ac, Bc: Higher magnifications of the highlighted square in panels Ab and Bb. Ad, Ae, Bd, Be: Bright-field images of the same areas as Ab, Ac, Bb, and Bc, respectively. The GAD 65/67-positive cells were stained with black. White and black arrows indicate the recorded neuron. Af, Bf: Membrane potential evoked by electrical stimulation to the T3 ventral root. Five trials were overlapped.

In seven of the 23 inspiratory cells, the electrical stimulation to T3VR caused the excitatory postsynaptic potential. We examined the existence of GAD65/67 mRNA in five cells using a mixed GAD65/67 probe; four of the cells were GAD65/67-positive. The data obtained from two GAD-positive cells with contrasting firing characteristics are shown in Fig. 4. The cell shown in Fig. 4A fired 0–2 times per inspiratory phase (0.7 ± 0.6), whereas the cell shown in Fig. 4B fired 4–14 times per inspiratory phase (9.6 ± 2.7). We examined the existence of GLYT2 in one Renshaw cell; it was positive. The anatomical characteristics were not significantly different between the Renshaw cells and the inspiratory interneurons (Table 1). Similarly, neither the firing characteristics nor the resting membrane potentials were significantly different (Table 1).

### Distribution of the inspiratory interneurons in the transverse plane

**Fig. 5:**
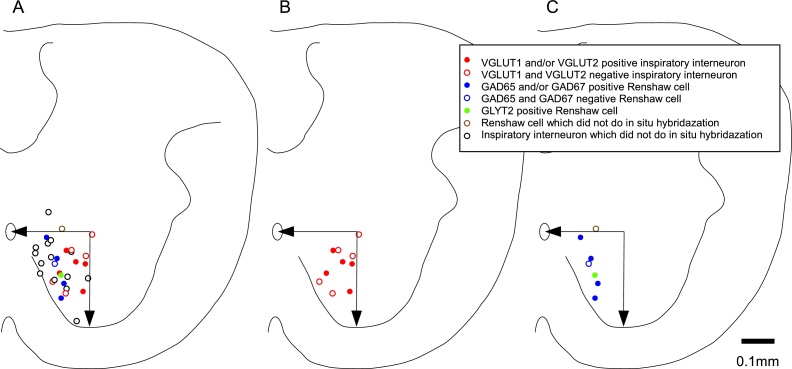
The distribution of the recorded neurons in the cross-sectional plane. A: All recorded cells are plotted. The positions of the inspiratory interneurons obtained in the preliminary experiment were also plotted (black circles). B: VGLUT-positive and -negative inspiratory interneurons are plotted. C: GAD65/67-positive and -negative, and GLYT2-positive Renshaw cells are plotted. Patch electrode penetrations were attempted only in the ventromedial region indicated by arrows in panels A–C.

The distribution of the inspiratory interneurons and the Renshaw cells in the transverse plane is illustrated in Fig. 5. In the present study we inserted the electrode into the medio-ventral side of the spinal cord. In this explored area, the distribution of the VGLUT-positive neurons overlapped with the area of the VGLUT-negative neurons. Similarly, the distribution area of the Renshaw cells overlapped with the area of the VGLUT-positive and -negative neurons.

## Discussion

### Excitatory inspiratory interneurons

Our results indicate that some inspiratory interneurons in the ventromedial region of the neonatal rat upper thoracic spinal cord expressed mRNA of vesicular glutamate transporters 1 and/or 2 (VGLUT), and thus these are glutamatergic excitatory neurons. Although several studies have described many inspiratory interneurons in the spinal cord (Kirkwood et al., 1988, 1993; Schmid et al., 1993; Saywell et al., 2011), the present study is the first to demonstrate that some inspiratory interneurons are glutamatergic. The interneurons projecting to the thoracic ventral horn are distributed mainly in the contralateral medial ventral horn in the same spinal segment (Schmid et al., 1993). Kirkwood et al. (1993) demonstrated that all five of the strongly modulated phasic inspiratory interneurons in the medial ventral horn showed positive-going focal synaptic potentials, indicating that these neurons are inhibitory, while six out of eight tonic interneurons only partly modulated with inspiration showed negative-going focal synaptic potentials.

Although the reason for this difference between Kirkwood et al. (1993) and our present findings is unknown, there are several differences between the two studies that should be considered. One is that the recording was obtained at the sixth or seventh thoracic segments in Kirkwood et al. (1993), but at third thoracic segment in the present study. Adult cats were used in Kirkwood et al. (1993); we used neonatal rats. Since it has been shown that monosynaptic projections from the bulbospinal inspiratory neurons to the inspiratory external intercostal motoneurons are rare (Merrill and Lipski, 1987), excitatory inspiratory interneurons should exist. The glutamatergic inspiratory interneurons demonstrated in the present study would be one of the candidates that convey the excitatory inspiratory input to the intercostal inspiratory motoneurons.

We observed herein that five of the 11 neurons were VGLUT-negative inspiratory interneurons. While it is known that small cholinergic interneurons are clustered around the central canal in the central gray matter (Barber et al., 1984), there are no cholinergic interneurons in the area we explored in this study. There are GABAergic and glycinergic interneurons in this area (Nishimaru et al., 2005, 2006; Restrepo et al., 2009), and it thus is possible that these interneurons are inhibitory.

Based on our previous findings that the inspiratory optical signals are larger in the thoracic segments at more rostral sites and that near-midline sites did not exhibit motoneuronal signals (Iizuka et al., 2016), in the present study we inserted the electrode only into the ventromedial side of the thoracic cord. In this explored area, the distribution area of the VGLUT-positive neurons overlapped with the area of the VGLUT-negative neurons. Similarly, the VGLUT-positive and -negative inspiratory cells and Renshaw cells intermingled with each other. This situation seemed to be similar to that of thoracic motoneurons. Thus, despite functional differences between the internal and external intercostal motoneurons, these motoneurons have similar morphological characteristics (Lane, 2011).

Assessments of the somatotopic distribution of each motoneuron type have shown some intermingling within the ventral horn, but to varying degrees (Larnicol et al., 1982; Rikard-Bell et al., 1985; Lipski and Martin-Body, 1987). Since the thoracic motoneurons form longitudinal columns of cells over the whole thoracic spinal cord (Lane, 2011), localizing specific types of interneurons may be inefficient to control the activity of thoracic motoneurons scattered in the whole segments.

The area examined in the present study corresponded to the medial part of lamina VII and VIII. Previous studies have indicated that the last-order interneurons to lumbar or cervical motoneurons are distributed in lamina V–VII of the ipsilateral side, and in lamina VIII of the contralateral side (Kitazawa et al., 1993; Coulon et al., 2011). Therefore, many of the recorded cells in the present study could be commissural interneurons. The commissural interneurons in the lumbar spinal cord have been extensively studied since commissural interneurons play an important role in the coordination of left/right alternation during locomotion (Birinyi et al., 2003; Quinlan and Kiehn, 2007). Using spike-triggered averaging of the lumbar ventral root potentials, Quinlan and Kiehn (2007) showed that the majority of the commissural interneurons gave inhibitory connections to motoneurons (36 of 43 interneurons), and an excitatory connection was identified in only seven cells. This connection will be favorable to organize left/right alternation during locomotion.

Quinlan and Kiehn (2007) also showed that both monosynaptic excitatory and inhibitory synaptic connection to contralateral motoneurons exist. Although there is no direct evidence showing a direct connection from the commissural inspiratory interneurons to thoracic inspiratory motoneurons, Kirkwood et al. (1993) reported that the strongly modulated phasic inspiratory interneurons showed positive-going focal synaptic potential in the contralateral ventral horn, indicating that these neurons were inhibitory. Therefore, the main role of the inspiratory interneurons might be inhibitory shaping or a suppressive control of inspiratory motor output.

In the present study, approximately one-half of the interneurons were glutamatergic. Since about one-third of total recorded cells were Renshaw cells, a total of about one-third of the cells can be expected to be glutamatergic. These glutamatergic interneurons could have excitatory influence on the inspiratory motoneurons. Respiratory interneurons are distributed over the entire ventral side of the spinal cord (Saywell et al., 2011). Qin et al. (2002) showed that there are many respiratory interneurons in the intermediate zone of the third thoracic cord. These respiratory interneurons receive and integrate noxious somatic and visceral information and would modulate the respiratory motor output (Qin et al., 2002). Further experiments should be conducted to clarify the distribution of the VGLUT-positive inspiratory interneurons in other areas including the motoneuron pool, and to examine the existence of the direct excitatory connection to inspiratory motoneurons.

Our present findings showed that the inspiratory interneurons in the ventromedial T3 segment were generally small in size compared to previous descriptions of the medullary respiratory neurons in neonatal rats (Onimaru and Homma, 1992; Ballanyi et al., 2009). However, one of these previous studies revealed that some types of medullary inspiratory interneurons have sizes that are comparable to those of the spinal inspiratory interneurons in the present study (Onimaru and Homma, 1992).

Input resistance is also one of the parameters indicating the size of recorded cells. In one study recorded from the ventrolateral medulla, the mean input resistance of the inspiratory neuron was 306 ± 104 MΩ (Onimaru and Homma, 1992). In another study of neonatal rat preparations, the input resistance of the medullary inspiratory neurons was 429 ± 79 MΩ (Ballanyi et al., 2009). In the present study, on the other hand, the mean input resistance was 598 ± 274 MΩ, suggesting poor development of the dendrites. The finding that many inspiratory interneurons are small may indicate that the small size is beneficial for precise motor control.

### Renshaw cells

Renshaw cells were uniquely identified as receiving ventral root-evoked short-latency excitatory postsynaptic potentials that were markedly reduced in amplitude by nicotinic receptor blockers (Mentis et al., 2005; Nishimaru et al., 2005, 2006). It has been shown that Renshaw cells express GABA, like most other inhibitory neurons in the spinal cord at the neonatal period (Nishimaru et al., 2005). In accordance with that study, most of the Renshaw cells identified in our present investigation were GAD65/67-positive. Therefore, most of the Renshaw cells in the experiments described herein can be considered true Renshaw cells.

We also observed that one of one Renshaw cell was GLYT2-positive, and one of five Renshaw cells was GAD65/67-negative. Two possibilities could be considered regarding the GAD65/67-negative cell; one is that the cell was pure glycinergic, and the other is that the cell was excitatory. A recent immunohistochemical study showed that in neonatal mice, GLYT2 and GAD are often colocalized in the same terminals in the ventral horn of the spinal cord; during the second postnatal week, GABAergic terminals markedly decreased and glycinergic terminals became dominant (Sunagawa et al., 2017). Although terminals that were GLYT2-positive but GAD-negative were sparse at postnatal day 0 (Sunagawa et al., 2017), it is still possible that the GAD65/67-negative cell observed in the present study was pure glycinergic. Related to the possibility that the GAD65/67-negative cell was excitatory, some studies suggest that there are recurrent excitatory pathways from the recurrent collaterals of motoneurons (Machacek and Hochman, 2006; Bonnot et al., 2009).

A recent study showed that there is recurrent excitation between motoneurons in the mouse lumbar spinal cord (Bhumbra and Beato, 2018). Since the spinal cord was cut at the level between T3 and T4 ventral roots, and the patch electrode was inserted from the cut surface (Fig. 1), it might be possible that T4 motoneurons were accidentally mixed in some of Renshaw cells and inspiratory interneurons in the present study. Since the electrode was inserted only to the ventromedial region of the spinal cord, the recording from T3 motoneuron was rare as described. However, we experienced two T3 motoneurons positioned in the ventromedial region (data not shown). We also recorded from three T3 motoneurons in the ventrolateral region. The mean input resistance of these motoneurons was 181.0 ± 27.6 MΩ (n = 5), and this value was quite lower than those of the Renshaw cells and the inspiratory interneurons (Table 1). Therefore, it is unlikely that the recordings obtained from T4 motoneurons were mixed in the present study.

The Renshaw cells in the phrenic motor nucleus receive non-cholinergic excitatory central respiratory input (Hilaire et al., 1986). It has been shown that the Renshaw cells in the lumbar spinal cord receive locomotor-like excitatory and inhibitory synaptic input from the commissural interneurons (Nishimaru et al., 2006). Thus, Renshaw cells generally could receive not only the excitatory synaptic inputs from the axon collaterals of motoneurons but also from central neuronal networks to regulate the motor outputs. Although the present and previous studies' findings showed that there are inspiratory Renshaw cells in the thoracic spinal cord (Kirkwood et al., 1981; Saywell et al., 2013), it remains unknown whether these Renshaw cells receive the central respiratory input. Further experiments are necessary to examine the existence and strength of the central respiratory input to elucidate the role of Renshaw cells in the regulation of the inspiratory motor outputs.

It is well documented that Renshaw cells are excited by axon collaterals from motoneurons and provide recurrent inhibition of synergistic motoneurons (Renshaw, 1941; Eccles et al., 1954). This recurrent inhibition has been observed at all spinal levels, i.e., in the cervical (Lipski et al., 1985; Brink and Suzuki, 1987), thoracic (Kirkwood et al., 1981; Saywell et al., 2013), lumbar (Renshaw, 1941; Eccles et al., 1954), and sacral segments (Jankowska et al., 1978). The mapping of the Renshaw cells in the sagittal plane showed the cells distributed at the ventral area of laminar VII, and thus at a slightly medial position of the motoneuron pool in the cat lumbar cord (Fyffe, 1990).

Similarly, in neonatal mouse, the Renshaw cells were distributed in the medioventral position of the lumbar cord (Gonzalez-Forero and Alvarez, 2005; Moore et al., 2015). As far as we know, however, the precise distribution of Renshaw cells in the sagittal plane of the cervical, thoracic and sacral spinal cord is not available. Recordings from Renshaw cells have been described only in the vicinity of the phrenic nucleus (Lipski et al., 1985), within the ventral horn of the thoracic spinal cord (Kirkwood et al., 1981). Although systematic tracking is necessary, our present experiments demonstrated the existence of Renshaw cells in the ventromedial area of the neonatal rat thoracic spinal cord.

A characteristic of Renshaw cells in adult mammals is a repetitive burst discharge followed by motor nerve stimulation (Renshaw, 1941; Eccles et al., 1954; Kirkwood et al., 1981). In the present study, most of the cells fired once when the ventral root was electrically stimulated. In neonatal mouse, high-frequency firing of Renshaw cells was observed in the lumbar spinal cord (Mentis et al., 2005; Nishimaru et al., 2006). However, Fig. 1b and Fig. 2d,e of Nishimaru et al. (2005) are examples of single firing followed by ventral root stimulation. Therefore, single firing followed by ventral stimulation could be a characteristic of the neonatal rat and indicate immaturity.

In conclusion, the present study showed for the first time that some inspiratory interneurons in the ventral horn of the neonatal rat upper thoracic spinal cord are glutamatergic, and these interneurons are one of the candidates that may enhance the inspiratory intercostal motor activity.

## Conflicts of interest

The authors have no conflicts of interest.
